# Evolutionary dynamics of the accessory genomes of *Staphylococcus aureus*

**DOI:** 10.1128/msphere.00751-23

**Published:** 2024-03-19

**Authors:** Kathryn R. Piper, Odion O. Ikhimiukor, Stephanie S. R. Souza, Teddy Garcia-Aroca, Cheryl P. Andam

**Affiliations:** 1 Department of Biological Sciences, University at Albany, State University of New York, Albany, New York, USA; 2 Department of Plant Pathology, University of Nebraska-Lincoln, Lincoln, Nebraska, USA; Escola Paulista de Medicina/Universidade Federal de São Paulo, São Paulo, Brazil

**Keywords:** *Staphylococcus aureus*, accessory genome, pan-genome, evolution, antimicrobial resistance

## Abstract

*Staphylococcus aureus* is a ubiquitous commensal and opportunistic bacterial pathogen that can cause a wide gamut of infections, which are exacerbated by the presence of multidrug-resistant and methicillin-resistant *S. aureus*. *S. aureus* is genetically heterogeneous and consists of numerous distinct lineages. Using 558 complete genomes of *S. aureus*, we aim to determine how the accessory genome content among phylogenetic lineages of *S. aureus* is structured and has evolved. Bayesian hierarchical clustering identified 10 sequence clusters, of which seven contained major sequence types (ST 1, 5, 8, 30, 59, 239, and 398). The seven sequence clusters differed in their accessory gene content, including genes associated with antimicrobial resistance and virulence. Focusing on the two largest clusters, BAPS8 and BAPS10, and each consisting mostly of ST5 and ST8, respectively, we found that the structure and connected components in the co-occurrence networks of accessory genomes varied between them. These differences are explained, in part, by the variation in the rates at which the two sequence clusters gained and lost accessory genes, with the highest rate of gene accumulation occurring recently in their evolutionary histories. We also identified a divergent group within BAPS10 that has experienced high gene gain and loss early in its history. Together, our results show highly variable and dynamic accessory genomes in *S. aureus* that are structured by the history of the specific lineages that carry them.

**IMPORTANCE:**

*Staphylococcus aureus* is an opportunistic, multi-host pathogen that can cause a variety of benign and life-threatening infections. Our results revealed considerable differences in the structure and evolution of the accessory genomes of major lineages within *S. aureus*. Such genomic variation within a species can have important implications on disease epidemiology, pathogenesis of infection, and interactions with the vertebrate host. Our findings provide important insights into the underlying genetic basis for the success of *S. aureus* as a highly adaptable and resistant pathogen, which will inform current efforts to control and treat staphylococcal diseases.

## INTRODUCTION

*Staphylococcus aureus* is a ubiquitous commensal and opportunistic bacterial pathogen that can cause a wide gamut of infections in humans and vertebrate animals (1). Disease manifestations in humans range from relatively benign abscess, impetigo, and cellulitis to more severe and life-threatening invasive diseases such as septicemia, osteomyelitis, endocarditis, and toxic shock syndrome (2). Particularly concerning is the spread of multidrug resistance (MDR) and methicillin-resistant *S. aureus* (MRSA), which further complicates and limits available treatment options (3). The public health burden and health care costs associated with *S. aureus* infections are staggering (4–6). In the most recent study of global burden of disease of 33 bacterial pathogens carried out in 2019, *S. aureus* was the leading bacterial cause of death in 135 countries (1,105,000 deaths, range: 816,000–1,470,000) and ranked second highest in terms of the years of life lost (34.3 million, range 25.5–45.3) (6). It is also the leading cause of fatal bloodstream infections with 299,000 deaths (range: 166,000–485,000) (6).

*S. aureus* can be differentiated into lineages according to sequence-based molecular methods. Sequence types (ST) and the clonal complexes (CC), formed from closely related STs, vary in their phenotypic and epidemiological characteristics, including the propensity to cause outbreaks and epidemics (7). The prevalence of individual lineages may vary, depending on geography, identity of vertebrate hosts, and disease manifestations (8–11). As of October 2023, there are 8,700 recognized STs in the *S. aureus* database of pubMLST (12). Of these, >5,700 STs are classified into 10 major CCs, whereas the rest remain poorly studied or unknown. Despite such tremendous diversity, only a few MRSA clones have instigated pandemic proportions of diseases (7). The most common nosocomial MRSA lineages are ST5 (CC5), ST8 and ST239 (both from CC8), ST22 (CC22), ST36 (CC30), and ST545 (CC45) (13). Accessory genes, that is, genes carried by a subset of a group of genomes and are not essential to every strain of a species (14, 15), provide a clue to the success of these lineages. Accessory genes encode antibiotic resistance, virulence, defense, or immune evasion mechanisms and are often carried by mobile genetic elements, such as plasmids, transposons, chromosomal cassettes, prophages, and pathogenicity islands (16, 17). Studies of the pan-genome, that is, the totality of genes in a set of strains (18), of *S. aureus* reveal significant variability in the accessory genomes within the species, but the processes that gave rise to lineage-specific pan-genomes remain unclear. Such fine-scale resolution in genomic variability within a species was recently termed comparative pan-genomics (19). In that study, the authors revealed the non-uniformity in ST-specific pan-genomes in *Escherichia coli*, which may reflect the species’ ecological flexibility (19). The evolutionary (e.g., selection versus neutral evolution), genetic (e.g., restriction-modification systems), and ecological drivers that shape the accessory gene content of specific microbial species remain a lively area of investigation (20–27).

Here, we aim to determine how the accessory genomes among *S. aureus* lineages are structured and have evolved. Analysis of 558 complete genomes of *S. aureus* showed considerable heterogeneity in gene content, gene co-occurrence, and rates of gene gain and loss in the accessory genomes between lineages. Variable accessory genomes within each lineage have important implications on bacterial physiology, pathogenesis of infection, and interactions with the vertebrate host. Our findings are critical in understanding the genetic basis for the success of *S. aureus* as a highly adaptable and resistant pathogen.

## RESULTS

### Phylogenetic relationships of *S. aureus* lineages

**Fig 1:**
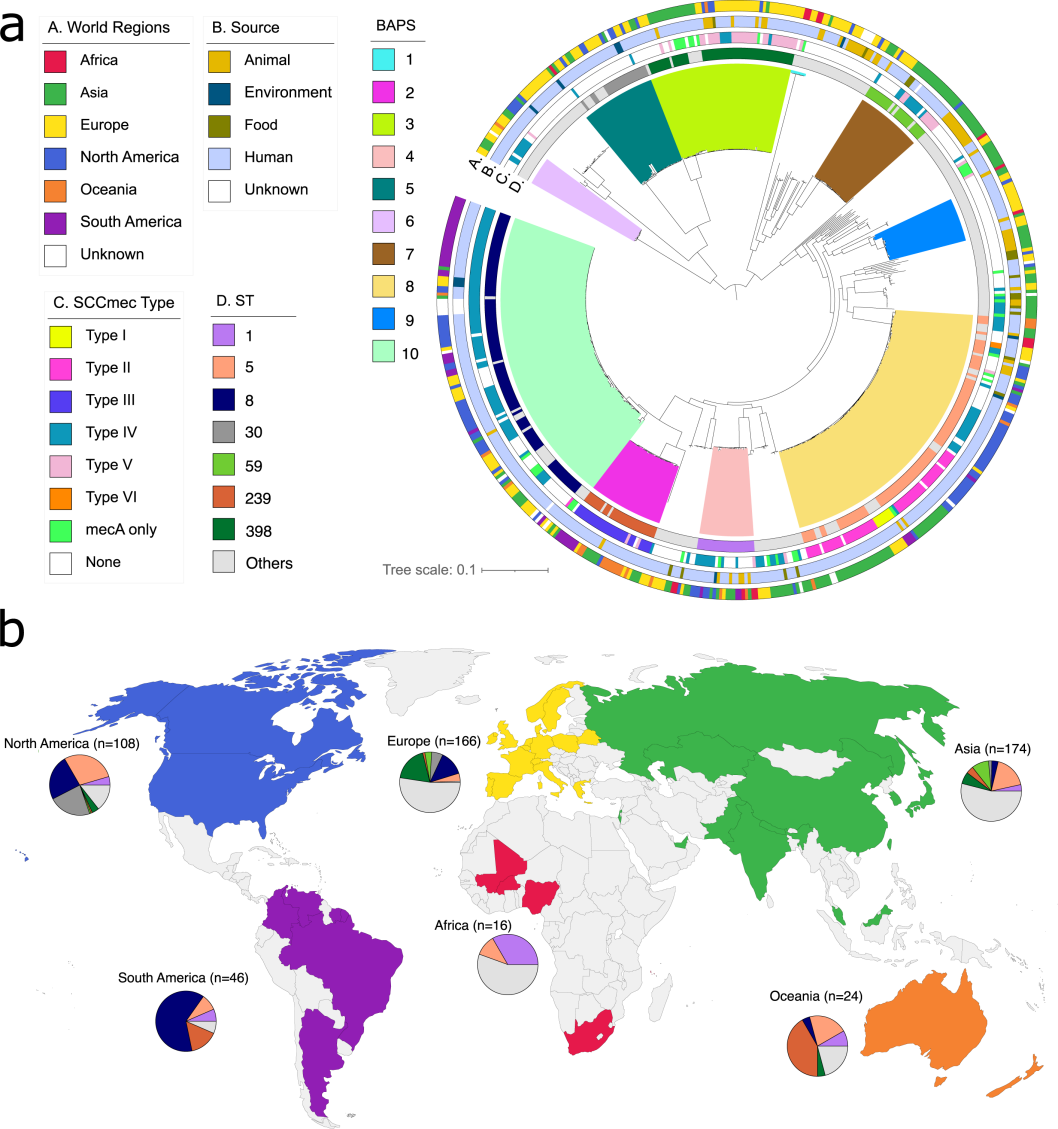
Phylogenetic relationship and geographical distribution of the 558 *S*. *aureus* used in this study. (**a**) Midpoint-rooted maximum likelihood tree showing the phylogenetic relationships of 558 *S*. *aureus* genomes. The tree was built using 235,221 SNPs of aligned sequences of 2,104 core genes and 53 soft core genes. Tree scale represents the number of nucleotide substitutions per site. Colored lines extending out of the branches in the tree represent the 10 sequence clusters inferred by the program fastBAPS. The four outer rings represent the geographical source (continent), ecological source, SCC*mec* type, and sequence type (ST). For visual clarity, only the seven major STs are shown. (**b**) World map showing the countries and continents from where the 558 *S*. *aureus* was derived. The pie charts show the distribution of the seven major STs per continent. The number above each pie chart shows the number of genomes per continent.

We obtained a total of 558 *S*. *aureus* complete genomes from the National Center for Biotechnology Information (NCBI). The strains were collected from 1884 to 2022 from 45 countries in six continents (Fig. 1a; Fig. S1; Table S1). Most genomes came from Asia (*n* = 174), Europe (*n* = 166), and North America (*n* = 108) (Fig. 1b). Most of the strains were derived from human sources (*n* = 453), whereas the rest came from food, environment, and water sources (Fig. 1a). Genome sizes ranged from 2.66 Mb – 3.13 Mb. The number of predicted genes ranged from 2,371 to 2,937 per genome. The pan-genome contained 2,104 core genes (present in 553–558 genomes), 53 soft core genes (present in 531–552 genomes), 826 shell genes (present in 84–530 genomes), and 5,020 cloud genes (present in 1–83 genomes) for a total of 8,003 genes (Table S2). The combined core and soft core made up 26.95% of the pan-genome, and the shell and cloud genes together comprised 73.05% of the pan-genome. A total of 1,557 genes were unique to a single genome.

Multi-locus sequence typing (MLST) analysis revealed the presence of 110 previously recognized ST (Fig. 1a; Table S3). Seven STs (STs 1, 5, 8, 30, 59, 239, and 398) together comprised 58.6% of the genomes in the data set. The most prevalent STs were ST8 (99/558 genomes representing 17.7% of the population) and ST5 (89/558 genomes or 15.9%). Other well-represented STs were ST398 (*n* = 48 genomes), ST239 (*n* = 27), ST59 (*n* = 23), ST1 (*n* = 21), and ST30 (*n* = 20). The remaining STs consisted of <20 genomes. We identified 67 STs that were represented by only a single genome.

The maximum likelihood phylogenetic tree built from 235,221 single nucleotide polymorphisms (SNPs) derived from sequence alignment of the combined 2,104 core and 53 soft core genes revealed deep branching monophyletic lineages (Fig. 1a). These lineages corresponded to 10 sequence clusters inferred by Bayesian hierarchical clustering (28) that groups together genetically similar sequences. The largest sequence cluster BAPS10 consisted of 116 genomes representing 8 STs (99 genomes from ST8, 7 genomes from ST254, 4 genomes from ST630, 2 genomes from ST923, and 1 genome each from ST507, ST612, ST1516, and ST5863). Another large cluster is BAPS8, which contained 113 genomes representing 14 STs (89 genomes from ST5, 5 genomes from ST228, 4 genomes from ST764, 2 genomes each from ST105, ST965, ST2389, and ST7358, and 1 genome each from ST587, ST632, ST950, ST5286, ST5352, ST6313, and ST6324). The remaining sequence clusters consisted of fewer genomes and were largely associated with one ST: ST152 in BAPS1 (*n* = 5 of 6 genomes), ST239 in BAPS2 (*n* = 27/29), ST398 in BAPS3 (*n* = 48/55), ST1 in BAPS4 (*n* = 21/21), ST30 in BAPS5 (*n* = 20/29), ST22 in BAPS6 (*n* = 11/11), ST59 in BAPS7 (*n* = 23/26), and ST15 in BAPS9 (*n* = 13/17).

### Antimicrobial resistance (AMR) and virulence genes in the accessory genomes

**Fig 2:**
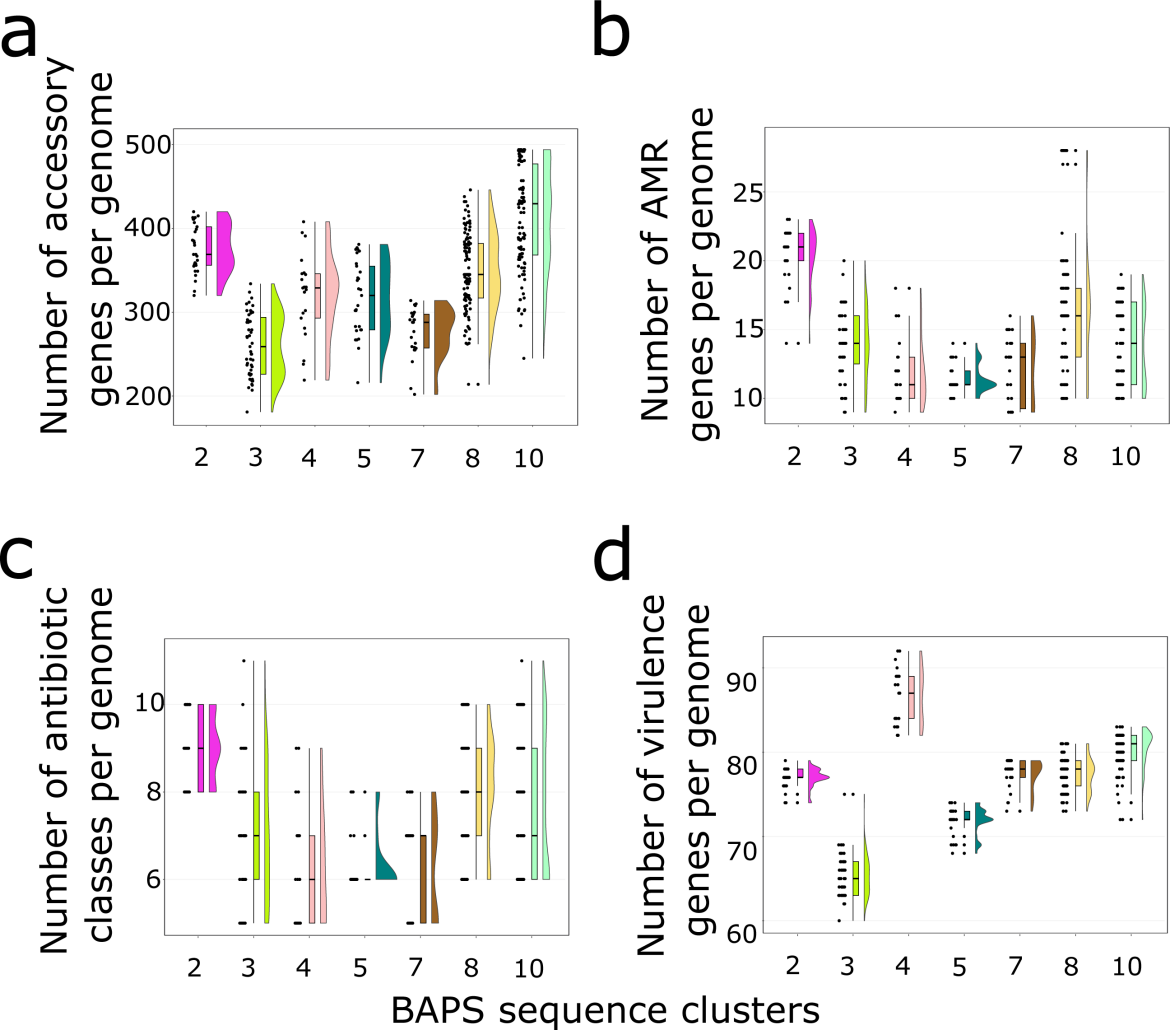
Comparison of the accessory genomes among the seven BAPS sequence clusters. Only sequence clusters with ≥20 genomes were compared. (**a**) Number of accessory genes per genome. (**b**) Number of acquired antimicrobial resistance genes per genome. (**c**) Number of antimicrobial classes that have at least one gene conferring resistance to an antimicrobial class present in a genome. (**d**) Number of virulence genes per genome. Box represents the interquartile range, horizontal line in the middle of the box represents the median, and the lower and upper whiskers represent the lowest data point without the outliers and the highest data point without outliers, respectively. For all panels, Welch’s *t*-test was used for all comparisons of every pair of sequence clusters.

We next sought to compare the accessory gene content of the sequence clusters with ≥20 genomes (BAPS 2, 3, 4, 5, 7, 8, and 10). In terms of the size of the accessory genome, sequence cluster BAPS10 carried the highest number of accessory genes per genome (mean = 413, range = 245–494) (Fig. 2a). Genomes from sequence cluster BAPS3 carried the lowest number of accessory genes per genome (mean = 260, range = 181–334). Comparison of every pair of sequence clusters showed significant differences in the number of accessory genes per genome (all comparisons with *P* value < 0.05; Welch’s *t*-test) (Fig. 2a).

Across the entire data set, we identified a total of 65 distinct genes associated with resistance to at least 16 antimicrobial classes (aminoglycoside, beta-lactam, diaminopyrimidine, fluoroquinolone, fusidame, glycopeptide, lincosamide, macrolide, mupirocin-like antibiotic, nucleoside, peptide, phenicol, phosphonic acid, pleuromutilin, streptogramin, and tetracycline) (Table S3). On average, genomes carried 13 AMR genes per genome (range = 9–28). Seven genes associated with AMR were present in all 558 genomes: *lmrS* (lincomycin resistance), *norA* (quinolone resistance), *arlRS* (regulate the expression of *norA*), *mepAR* (efflux protein), and *mgrA* (multidrug resistance). Among the seven major sequence clusters, genomes from BAPS2 (consisting mostly of ST239) carried the highest number of AMR genes per genome (mean = 20, range = 14–23), whereas genomes from BAPS5 carried the lowest number of AMR genes per genome (mean = 11, range = 10–14) (Fig. 2b). Pairwise comparisons of the seven major sequence clusters revealed significant differences in the number of AMR genes per genome (all comparisons with *P* value < 0.001; Welch’s *t*-test) (Fig. 2b) and in the number of antimicrobial classes that have at least one gene conferring resistance to an antimicrobial class present in a genome (all comparisons with *P* value < 0.01; Welch’s *t*-test) (Fig. 2c).

We also characterized the phylogenetic distribution of the mobile element staphylococcal chromosomal cassette *mec* (SCC*mec*) that carries the determinant for beta-lactam resistance encoded by the *mecA* gene (29). A total of 282 genomes (50.5% of the data set) carried the SCC*mec* (Fig. 1A; Table S3). SCC*mec* typing identified the presence of 6 SCC*mec* types and 13 subtypes: types I (*n* = 7 genomes), II (*n* = 50), III (*n* = 26), IV (*n* = 152), V (*n* = 38), and VI (*n* = 2). The majority of genomes in sequence cluster BAPS10 genomes carried SCC*mec* Type IV (77/116 genomes corresponding to 66.4%). A total of 49/113 genomes or (43.4%) in sequence cluster BAPS8 carried SCCmec Type II. A total of 32 genomes did not carry a SCC*mec* element (27.6%), and 6 genomes carried the *mecA* gene only (5.2%).

We identified a total of 97 unique virulence genes across the entire data set, of which 24 were present in all 558 genomes and have functions related to immune modulation (*adsA*), exoenzyme/exotoxin production (*aur, hlgBC, hld,* and *sspAC*), capsular polysaccharide synthesis (*cap8B, cap8C, cap8M, cap8O,* and *capAN*), type VII secretion system (*esaG*), adherence (*isdACDEFGI* and *srtB*), lipase activity (*geh*), and superantigen (*set25*) (Table S3). Comparing the seven major sequence clusters, we found significant differences in the number of virulence genes per genome (*P* values for all comparisons <0.001, Welch’s *t*-test) (Fig. 2d). Genomes from sequence cluster BAPS4 (consisting of ST1) carry the highest number of virulence genes per genome (mean = 86, range = 82–92). In contrast, genomes from sequence cluster BAPS3 carry the lowest number of virulence genes per genome (mean = 65, range = 60–75).

### Co-occurrence of accessory genes

**Fig 3:**
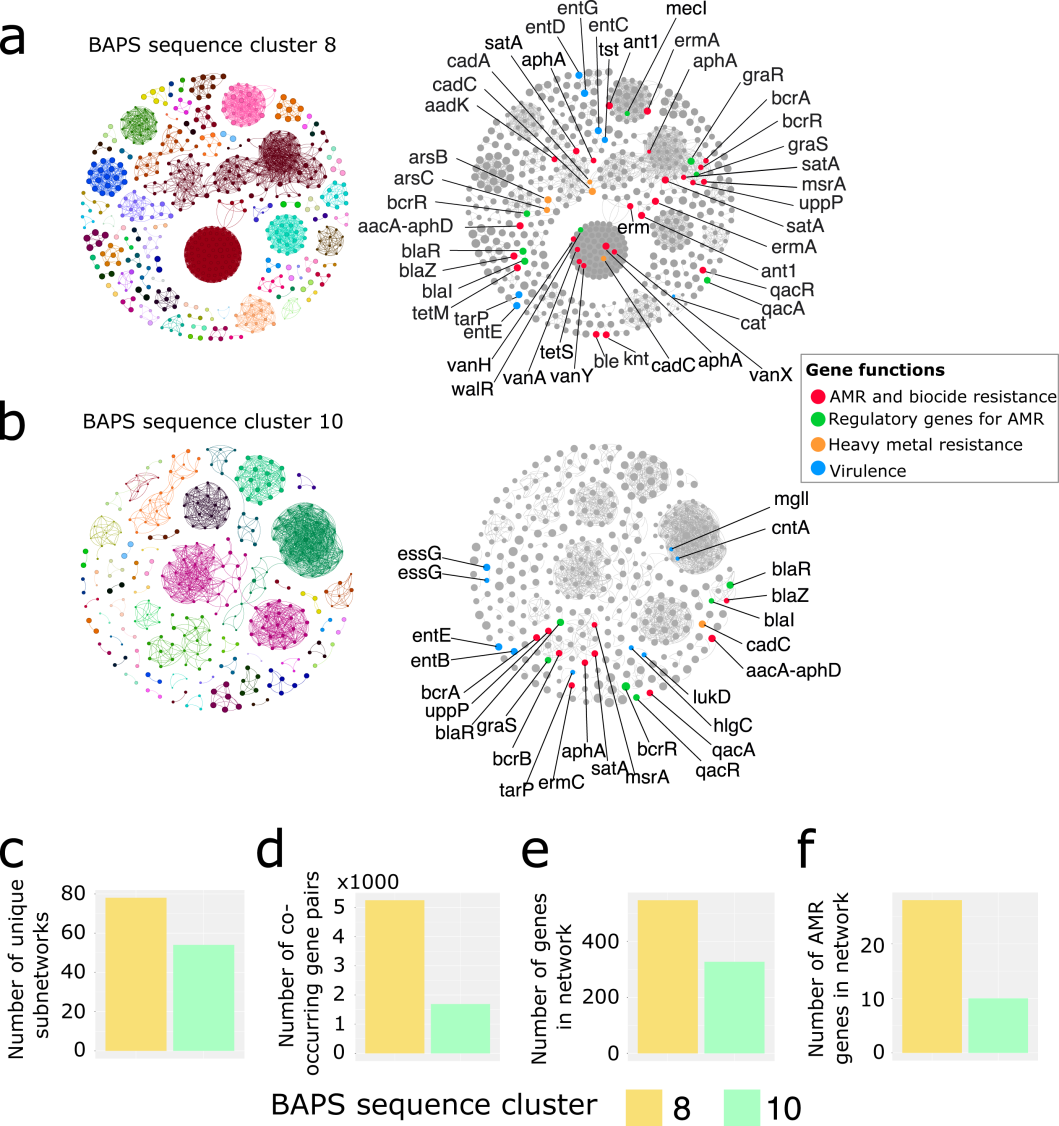
Co-occurrence networks of accessory genes between sequence clusters. Network diagrams created with Gephi using output from Coinfinder carried out on (**a**) 113 genomes in sequence cluster BAPS8 and (**b**) 116 genomes in sequence cluster BAPS10. Nodes represent specific accessory genes and edges that connect them represent significant gene association. On the left, subnetworks are shown in a unique color and contain nodes connected to each other. A subnetwork represents a subset of the larger network’s topology. On the right are networks identical to those shown on the left but with nodes color-coded according to gene functions (antimicrobial resistance, regulatory, heavy metal resistance, virulence). Comparison of the (**c**) number of unique subnetworks, (**d**) number of co-occurring gene pairs, (**e**) number of genes, and (**f**) number of AMR genes in each co-occurrence network built from sequence clusters BAPS8 and BAPS10.

Accessory genes may associate (or co-occur) with other genes more often than expected by chance (30). These co-occurring genes may reflect a shared evolutionary history, co-evolution of traits, correlated selection across environments, or coordinated patterns of gene gain or loss (30). We obtained statistically significant co-occurrence of accessory genes in the two largest sequence clusters BAPS8 and BAPS10 (Fig. 3) using Coinfinder (30). We also ran Coinfinder in the other eight sequence clusters; however, no significant gene associations were detected because they consisted of much fewer genomes, ranging from 6 to 55 genomes in each cluster. The number of gene associations detected by Coinfinder is known to decrease substantially with smaller numbers of genome inputs, with no significant associations identified when an input of around 50 genomes or fewer is used (30). For sequence cluster BAPS3 (*n* = 55 genomes), we postulated that the one divergent genome found at the base of the cluster (Accession no. GCF_003609855.1) may have contributed to the lack of significant gene associations in our analysis. We therefore ran Coinfinder on BAPS3 without this genome; however, we still did not obtain any significant gene associations.

In sequence cluster BAPS8 containing mostly ST5, we identified 5,249 statistically significant co-occurring gene pairs, which clustered into 78 connected components (subnetworks) in 547 gene families (Fig. 3a; Table S4). Within this network of interconnected genes were six virulence genes (*entC, entD, entE, entG, tarP,* and *tst*), 27 AMR resistance genes (*aacA-aphD, aadK, ant1, ant1, aphA, aphA, bcrA, ble, cat, erm, ermA, ermA, bcrB, satA, aphA, knt, satA, satA, tet(M), tet(S), uppP, msrA, vanA, vanH, vanX, vanY,* and *blaZ*), 7 regulatory genes for AMR (*blaR, blaI, bcrR, walR, mecI, graR,* and *graS*), five genes associated with heavy metal resistance (*arsB, asrC, cadA, cadC,* and *cadC*), and two genes associated with quaternary ammonium biocide resistance (*qacA* and *qacR*). The virulence genes in the network represent functions related to host immune system evasion and activation and toxic shock syndrome. The AMR genes in the network represent eight antimicrobial classes (aminoglycoside, beta-lactamase, glycopeptide, nucleoside, peptide, phenicol, streptogramin, and tetracycline). There were six subnetworks that contained co-occurring AMR genes.

In sequence cluster BAPS10 containing mostly ST8, we identified 1,688 co-occurring gene pairs, which clustered into 54 connected components (subnetworks) in 327 gene families (Fig. 3b; Table S5). Within this network of interconnected genes were eight virulence genes (*entB, entE, essG, essG, mgll, hlgC, lukD,* and *tarP*), nine AMR genes (*aacA-aphD, aphA, bcrA, bcrB, blaZ, ermC, satA, uppP,* and *msrA*), four regulatory genes for AMR (*blaR, blaR, blaI,* and *graS*), one gene associated with heavy metal resistance (*cadC*), and two genes associated with quaternary ammonium biocide resistance (*qacA* and *qacR*). The virulence genes in the network represent functions related to activating and evading the host immune system, inducing an inflammatory response, hemolytic and leucotoxic activity, and secretion. The AMR genes in the network represent five antimicrobial classes (aminoglycoside, beta-lactamase, nucleoside, peptide, and streptogramin). Only one subnetwork contained co-occurring AMR genes (*aphA, satA, bcrB, bcrA, uppP,* and *msrA*).

AMR genes that are common to both co-occurrence networks of BAPS8 and BAPS10 include *blaZRI* (beta-lactam resistance), *bcrABR* (bacitracin resistance), *qacA* (resistance to quaternary ammonium compounds), *satA* (resistance to nucleoside antibiotics [streptothricin]), *aphA* (aminoglycoside resistance), and *msrA* (resistance to streptogramin and macrolide). In both co-occurrence networks, there were 386 and 257 gene families that have hypothetical functions in sequence clusters BAPS8 and BAPS10, respectively. In terms of the structure of the co-occurrence networks, we found that the two sequence clusters BAPS8 and BAPS10 differed in terms of the number of unique subnetworks (Fig. 3c), number of co-occurring gene pairs (Fig. 3d), number of genes that form the co-occurrence network (Fig. 3e), and the number of AMR genes in the co-occurrence network (Fig. 3f).

### Rates of gene gain and gene loss

Accessory genes can be acquired or lost throughout the evolutionary history of a lineage. We therefore sought to determine the rates of gene gain and gene loss in sequence clusters along the branches of their respective phylogenies. Using the core SNP phylogenies, gene presence/absence matrices, and a generalized linear model implemented in Panstripe (31), we estimated the rates of gene gain and loss in the five major sequence clusters (BAPS2, BAPS3, BAPS5, BAPS8, and BAPS10). For BAPS3, we also calculated rates with and without the divergent genome (Accession no. GCF_003609855.1).

**Fig 4:**
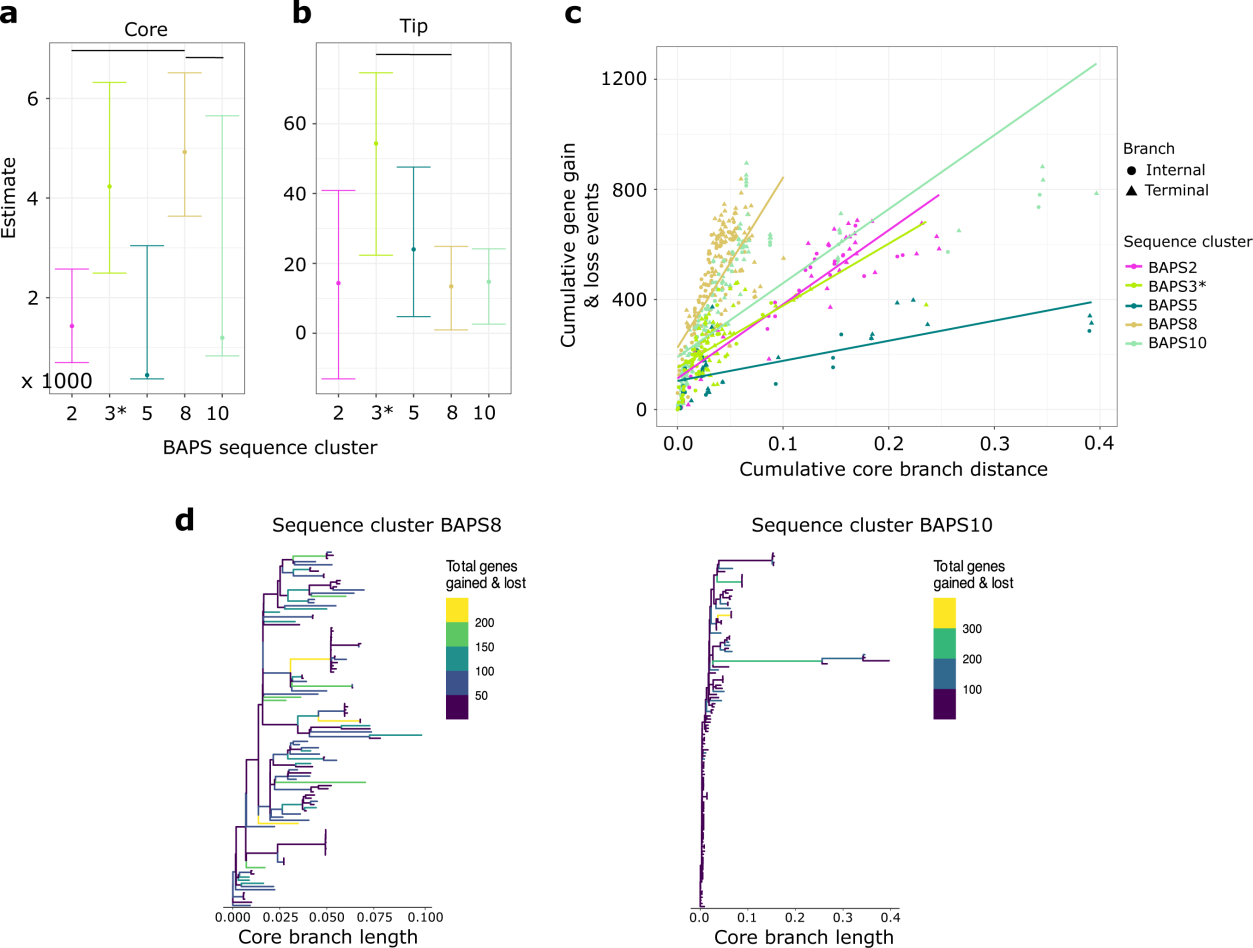
Rates of gene gain and gene loss between sequence clusters. (**a**) Association between the branch lengths in the core genome phylogeny and rates of gene gain and loss in sequence clusters BAPS2, BAPS3, BAPS5, BAPS8, and BAPS10. (**b**) Association between genes on the tips of the phylogeny and rates of gene gain and loss. For panels a and b, colored dots represent the mean and error bars represent the 95% bootstrap confidence intervals of the parameter estimates. (**c**) Cumulative gene gain and loss as a function of the cumulative core branch distance in the phylogeny. Internal and terminal branches are represented by circles and triangles, respectively. For panels a–c, calculation in BAPS3 was done without the divergent genome (Accession no. GCF_003609855.1) and is represented by an asterisk. (**d**) Inferred ancestral states showing the gene gain and loss events on phylogeny of sequence clusters BAPS8 (left) and BAPS10 (right). The branch colors show the total number of genes gained and lost along the internal and terminal branches of the trees. Phylogenies for sequence clusters BAPS2, BAPS3 (with and without the divergent genome), and BAPS5 are shown in Fig. S2.

We found higher rates of gene gain and loss in sequence cluster BAPS8 compared with BAPS10 (4925.19 and 1195.96, respectively, *P* value = 2.78e-10) measured as a function of the association between the branch lengths of the phylogeny and the number of gene gain and loss events (Fig. 4a). However, the pan-genomes of the two sequence clusters did not differ in the rates of gene gain and loss at the terminal branches of the phylogeny (13.42 and 14.74, respectively, *P* value = 6.81e-1; Fig. 4b). An elevated rate in the cumulative number of gene gain and loss events versus the cumulative branch length starting from the root node of their corresponding phylogenies can be detected for both sequence clusters (Fig. 4c). For both sequence clusters, we also found that the number of gene gain and loss events congregated in more recent branches of their respective phylogenies, suggesting frequent and rapid gene turnover at recent times (Fig. 4d). The enrichment for gene gain and loss events at the tips of a tree is often driven by the presence of highly mobile genetic elements and/or annotation errors (31). Within sequence cluster BAPS10, we identified a divergent group consisting of four genomes that has experienced high number of gene gain and loss early in the history of this sequence cluster, which was not observed in sequence cluster BAPS8.

We also carried out comparisons with other smaller clusters; however, the results should be considered with caution because of the considerably smaller number of genomes in them compared with BAPS8 and BAPS10. We found significant difference in the rates of gene gain and loss between sequence clusters BAPS2 and BAPS8 in their core genomes (1430.97 and 4925.19, respectively; *P* value = 1.09e-06) (Fig. 4a). Sequence clusters BAPS3 and BAPS8 (excluding the divergent genome in BAPS3) had significant differences in the rates of gene gain and loss at the terminal branches of their phylogenies (54.34 and 13.41, respectively; *P* value = 0.0362) (Fig. 4b). All other cluster comparisons were either not significant or failed in fitting the generalized linear model in Panstripe due to the low number of genomes in the smaller clusters (Table S6). The total number of genes gained or lost varied along each branch of the phylogenies of BAPS2, BAPS3, and BAPS5 (Fig. S2).

## DISCUSSION

The bacterial accessory genome consists of genes present in some but not all of the strains being considered (14, 15, 22). Because of its highly variable nature, how it has evolved and is structured (i.e., why some strains have certain genes and some do not) continues to be explored in microbial genomics. Using 558 complete genomes of *S. aureus*, we investigated the structure and evolution of the accessory genomes of the main lineages within the species. Our main findings are as follows: (i) the gene content and co-occurring components of the accessory genomes, including genes associated with AMR and virulence factors, varied between the major lineages. (ii) The two lineages differed in the rate at which they gained and lost accessory genes, with the highest rate of gene accumulation occurring late in each lineage’s evolutionary history. Together, our results indicate highly variable and dynamic accessory genomes in *S. aureus* that are structured by the history of the genetic lineages that carry them.

Considerable genomic variation within a species can have important implications on disease epidemiology, pathogenesis of infection, and strain interactions with the vertebrate host in many pathogens. An excellent example is a study whereby accessory gene content variation was used to track the transmission of extensive and multidrug-resistant *Acinetobacter baumannii* across hospital wards (32). Using accessory genomes in disease epidemiology proved to be effective because gene turnover (i.e., gain and loss) occurs faster than the accumulation of mutations in *A. baumannii* over very short microevolutionary scales (33). In *Streptococcus dysgalactiae*, the distinction between its two subspecies and their ability to adapt to either human or bovine host lie in a specialized accessory genome characterized by the presence of specific surface-protein virulence determinants and carbohydrate metabolic functions (34). In *S. aureus* from different vertebrate hosts, accessory gene pools consisting of host-specific genes promote adaptive evolution after host-switching events (10). Gain and loss of multiple accessory genes also promoted cattle specialization in *Campylobacter jejuni* ST61, which evolved from the host generalist lineage ST21 (35). Our results further expand on these previous studies and highlight the importance of lineage-specific accessory gene pools. Our results also show that the use of a single model or reference strain of a species, and even of an ST or any lineage, is not sufficient to capture the genomic diversity below the species level.

The accessory gene pool within a lineage of a species is highly heterogeneous not only in terms of gene functions but also the patterns of preservation and timing of mobility. In a previous study of *E. coli* ST131, the expansion of the accessory genomes of two clades C1 and C2 appears to have occurred more recently (31), which was similar to what we observed for the two *S*. *aureus* lineages in our study. Regardless of the timing of gene acquisition and loss, different mechanisms may act to preserve accessory genes: a more rapid mobilization occurring over short time scales and relatively slower transfer occurring over longer periods of time (16, 36). The types of mobile genetic elements and recombination processes will influence how slowly or rapidly accessory genomes are altered across a species or lineage. Hence, grouping all accessory genes as a single entity within the pan-genome may not fully capture the incredible variation in bacterial genomes. This is particularly evident for the divergent group within sequence cluster BAPS10 that experienced an enrichment of acquired and lost genes early in the evolutionary history of this lineage. The temporal analysis of the pan-genome is a powerful approach to studying how the pan-genome has evolved, which is not readily apparent in phylogenetic tree reconstruction and gene accumulation curves that are often used in microbial pan-genome investigations.

Our findings are consistent with results from a recent study that compared pan-genomes as highly dynamic ecosystems where genes recruited into individual genomes interact with other genes that they find themselves with either positively or negatively depending on the genetic background of the recipient genome (37). We further add that even if a gene has been acquired and lost multiple times throughout the history of a specific lineage, the recruited gene will likely find itself in a slightly different genetic neighborhood every time it is acquired due to the inconstancy in the presence of other genes within a genome of the lineage. Nonetheless, it remains challenging to distinguish repeated gain and loss of the same genes within a lineage. Although both genetic drift and selection shape the persistence and mobility of genes that comprise the pan-genome (37–39), the contributions of these processes vary over the time that a lineage has been evolving, and therefore, their impacts on pan-genome content over time will also fluctuate. Understanding the history and variability in the pan-genome within a species may therefore shed light on how ancestral lineages adapted to their environments and, in turn, how their environments shaped their genomes. As an example, the history of *S. aureus* is characterized by numerous instances of switching between humans and different animal hosts, and hence will certainly alter its pan-genome content (10). However, to what extent the genome content changes as a lineage switches from host species A to host species B compared with when it switches from host species A to host species C remains to be elucidated.

We acknowledge the limitations of our study. First, our data set is heavily favored toward genomes from certain geographical regions and from human clinical sources. Additional metadata (e.g., disease versus carriage and therapeutic history) were also not available. Hence, we were not able to make inferences about the ecological and geographical impacts to accessory genome structure. Second, our smaller data set (558 genomes) compared with other studies that number thousands of genomes may also limit the magnitude of accessory gene pool that we can identify. *S. aureus* is known to have an open pan-genome (40–42), that is, the pan-genome has no upper limit and new genes are discovered with the addition of new strains (18, 43). It is therefore likely that the co-occurrence networks and history of gene gain and loss will be affected with the addition of genomes and novel genes. Nonetheless, because we confined our analyses to complete genomes, we were able to demonstrate a more complete picture of the heterogeneity in structure and evolutionary history between the lineage-specific accessory genomes, which may be obscured when using draft and incomplete genomes. Our results have been largely confined to the two largest sequence clusters (BAPS8 and BAPS10), and hence, we can only make confident inferences about the evolutionary trajectories of their pan-genomes. Gene associations and historical rates of gene gain/loss remain difficult to infer in sequence clusters that consist of much fewer genomes. The range of diversity of missing relatives and missing branches on the phylogeny will inevitably lead to some genes remaining invisible in any pan-genome analysis and consequently accurately inferring the historical rates of gene gain and loss. Sequencing more genomes from less common lineages is expected to improve the predictive power of the approaches we used.

In summary, we show that the accessory genome of *S. aureus* is lineage-specific and is structured by the co-occurrence of certain genes and the histories of gene gain and loss. Our findings provide important fundamental insights into the underlying genetic basis for the highly adaptable, virulent, and multidrug resistance features of *S. aureus*, as well as the global success of certain *S. aureus* clones. This knowledge will inform current efforts to control and treat staphylococcal diseases, including approaches to target specific *S. aureus* clones or lineages.

## MATERIALS AND METHODS

### Genome collection and annotation

We retrieved a total of 888 complete genome sequences of *S. aureus* from the NCBI RefSeq Database in September 2022. Genome quality was assessed using the programs QUAST (44) and CheckM (45) (Table S1). Genomes with <90% completeness and >5% contamination were excluded from downstream analysis, as recommended by CheckM (45). All genomes were compared against each other using the program FastANI v.1.32 (46) to confirm species identification. We used the ≥95% average nucleotide identity (ANI) threshold to define a species (46). Genomes that did not meet the 95% criteria were also excluded. After filtering based on these criteria, we used a total of 558 genomes for all downstream analyses (Table S1). Our strict criteria for the inclusion of *S. aureus* genomes are critical to reduce the impact of missing genes in incomplete, draft, and poorly sequenced genomes. The genomes were annotated using Prokka v.1.14.6 (47).

### Pan-genome analysis and phylogenetic tree reconstruction

We used Panaroo v.1.2.7 (48) to determine the core and accessory gene content of the entire population. We defined core genes as those present in ≥95% of the genomes (i.e., at least 530 genomes), whereas the accessory genes were genes present in ≥1% and <95% of the data set (i.e., less than 530 genomes). Panaroo was run in strict mode with the –remove-invalid-genes option to ignore annotations that do not conform to the expected gene structure (i.e., with a premature stop codon or gene of an invalid length). Sequence alignment was carried out using MAFFT v.7.487 (49). Sequence alignments of 2,104 core genes and 53 soft core genes were concatenated, and SNPs were identified using SNP-site v.2.5.1 (50). A total of 235,221 SNPs were identified. The aligned core SNPs were used to build a maximum likelihood phylogenetic tree using RAxML v.8.2.12 (51) with a generalized time-reversible (GTR) (52) model of nucleotide substitution and Gamma distribution of rate heterogeneity. We used the Bayesian hierarchical clustering algorithm fastBAPS v.1.0.6 (fast Bayesian Analysis of Population Structure) to partition the genomes into sequence clusters consisting of genetically similar individuals (28). We also built core SNP phylogenetic trees for individual sequence clusters using the same methods described above.

### *In silico* identification of ST, AMR genes, virulence genes, and SCC*mec*

The ST was determined using the program mlst v.2.19.0 (https://github.com/tseemann/mlst), which extracts seven housekeeping genes (*arcC*, *aroE*, *glpF*, *gmk*, *pta*, *tpi,* and *yqiL*) (53) and compares allelic profiles against previously characterized STs in the *S. aureus* PubMLST database (12). We used ABRicate v1.0.1 (https://github.com/tseemann/abricate) to identify the presence of AMR and virulence genes. We used the minimum thresholds of >80% for sequence coverage and >80% sequence identity for comparing query sequences with the curated Comprehensive Antimicrobial Resistance Database (CARD) (54) and the Virulence Factor Database (VFDB) (55). We screened for the presence and type of *mecA*-carrying staphylococcal chromosomal cassette (SCC*mec*) using the stand-alone tool staphopia-sccmec v.1.0.0 (56).

### Identification of coincident resistance and accessory genes

To determine if AMR genes associate or dissociate with each other and with other accessory genes more often than would be expected by chance, we used the program Coinfinder v.1.0.7 (30). Coinfinder detects genes that associate or dissociate with other genes using a Bonferroni-corrected Binomial exact test statistic of the expected and observed rates of gene-gene association. Network diagrams of these coincident gene-to-gene relationship within each sequence cluster were visualized with Gephi v.0.9.2 (https://github.com/gephi/gephi). Genes associated with resistance and virulence were identified from the network clusters using output from ABRicate and gene functions listed in UniProt (57).

### Determining rates of gene gain and gene loss in the lineage pan-genomes

We ran Panstripe v.2.0 (31) to determine gene gain and gene loss estimates on the pan-genomes of individual sequence clusters. We used the core genome SNP phylogeny and the gene presence/absence matrix generated by Panaroo as input. Ancestral state reconstruction of gene gain and loss events on each branch was carried out using maximum parsimony implemented in Panstripe. Panstripe then takes the branch lengths in the phylogeny and compares them with the number of gene gain and loss events inferred to have occurred on that branch using a generalized linear model. We carried out bootstrapping with 1,000 replicates.

### Statistical analysis

We carried out all statistical analysis using the ggstatsplot v.0.12.1 package (58) in R v.4.3.1 (59). We used ANOVA and pairwise Welch’s *t*-test to compare the number of accessory genes per genome, number of AMR genes per genome, number of antibiotic classes per genome, and number of virulence genes per genome among the major STs. The results were considered significant when *p* < 0.05.

## SUPPLEMENTAL MATERIAL

Supplemental Figures msphere.00751-23-s0001.pdf Supplemental table captions and Fig. S1 and S2.

Supplemental Tables msphere.00751-23-s0002.xlsx Table S1-S6.

ASM does not own the copyrights to Supplemental Material that may be linked to, or accessed through, an article. The authors have granted ASM a non-exclusive, world-wide license to publish the Supplemental Material files. Please contact the corresponding author directly for reuse.
