# Supplementary material for: Evolutionary dynamics of the accessory genomes of Staphylococcus aureus
Source: mSphere. 2024 Mar 19;9(4):e00751-23. doi: 10.1128/msphere.00751-23 (PMC11036810; doi:10.1128/msphere.00751-23)
Supplement: Supplemental Figures — Supplemental table captions and Fig. S1 and S2. [file msphere.00751-23-s0001.pdf]

Evolutionary dynamics of the accessory genomes of *Staphylococcus aureus*

Kathryn R. Piper, Odion O. Ikhimiukor, Stephanie S.R. Souza, Teddy Garcia-Aroca, Cheryl P. Andam

Supplemental material

Supplementary Table S1. Accession numbers, associated metadata, and sequence clusters inferred by fastBAPS (fast Bayesian Analysis of Population Structure) of the 558 *S. aureus* genomes in this study.

Supplementary Table S2. List of all genes detected using Panaroo in the pan-genome of 558 *S. aureus*.

Supplementary Table S3. Distribution of acquired antimicrobial resistance genes, virulence genes, SCC*mec* type, and sequence type assignment of the 558 *S. aureus* genomes.

Supplementary Table S4. Co-occurring genes in sequence cluster BAPS8 inferred by Coinfinder.

Supplementary Table S5. Co-occurring genes in sequence cluster BAPS10 inferred by Coinfinder.

Supplementary Table S6. Rates of gene gain and loss estimated using Panstripe for every pairwise comparison of sequence clusters BAPS2,BAPS3,BAPS5, BAPS8, BAPS10

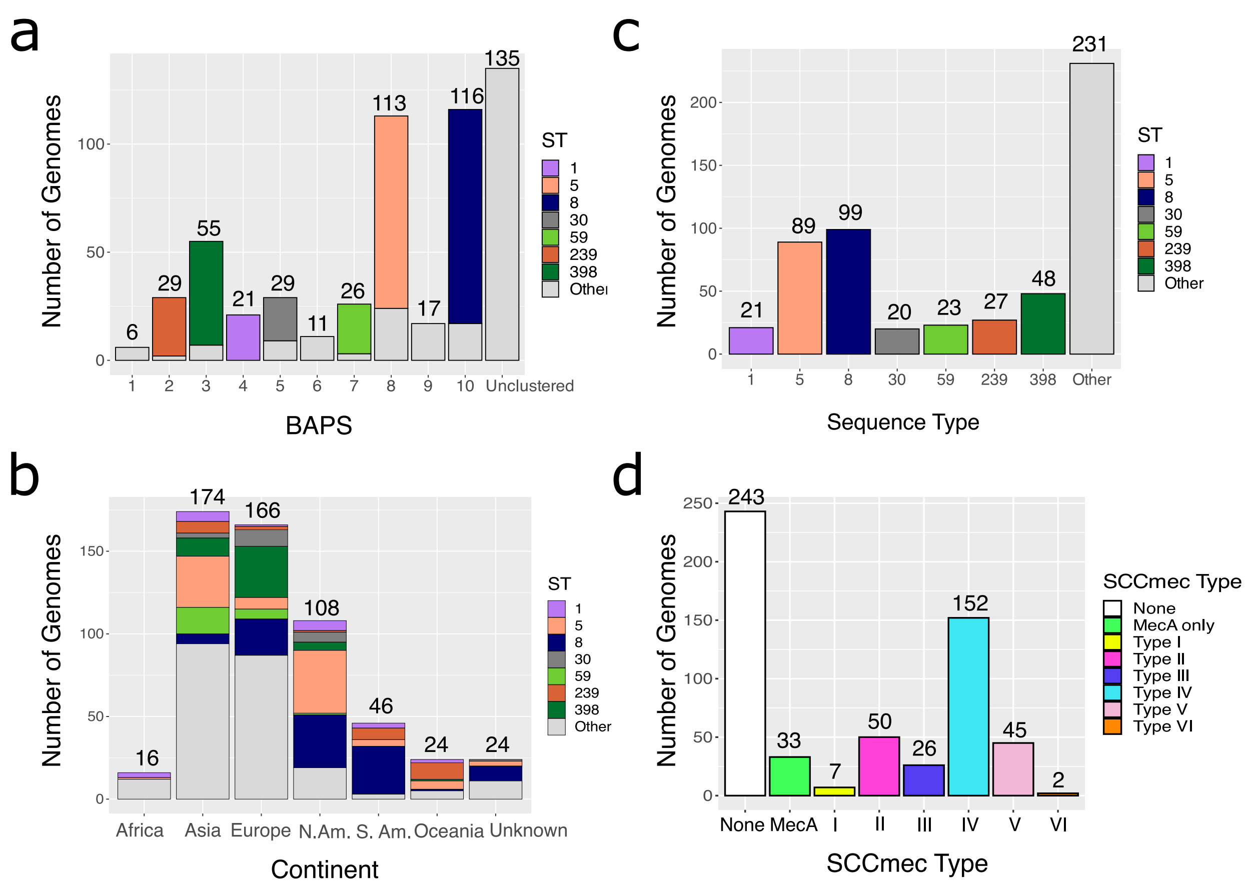

Supplementary Figure S1. Barplots showing the number and distribution of *S. aureus* genomes according to (a) BAPS sequence cluster, (b) sequence type (c) geographical region, and (d) SCC*mec* type.

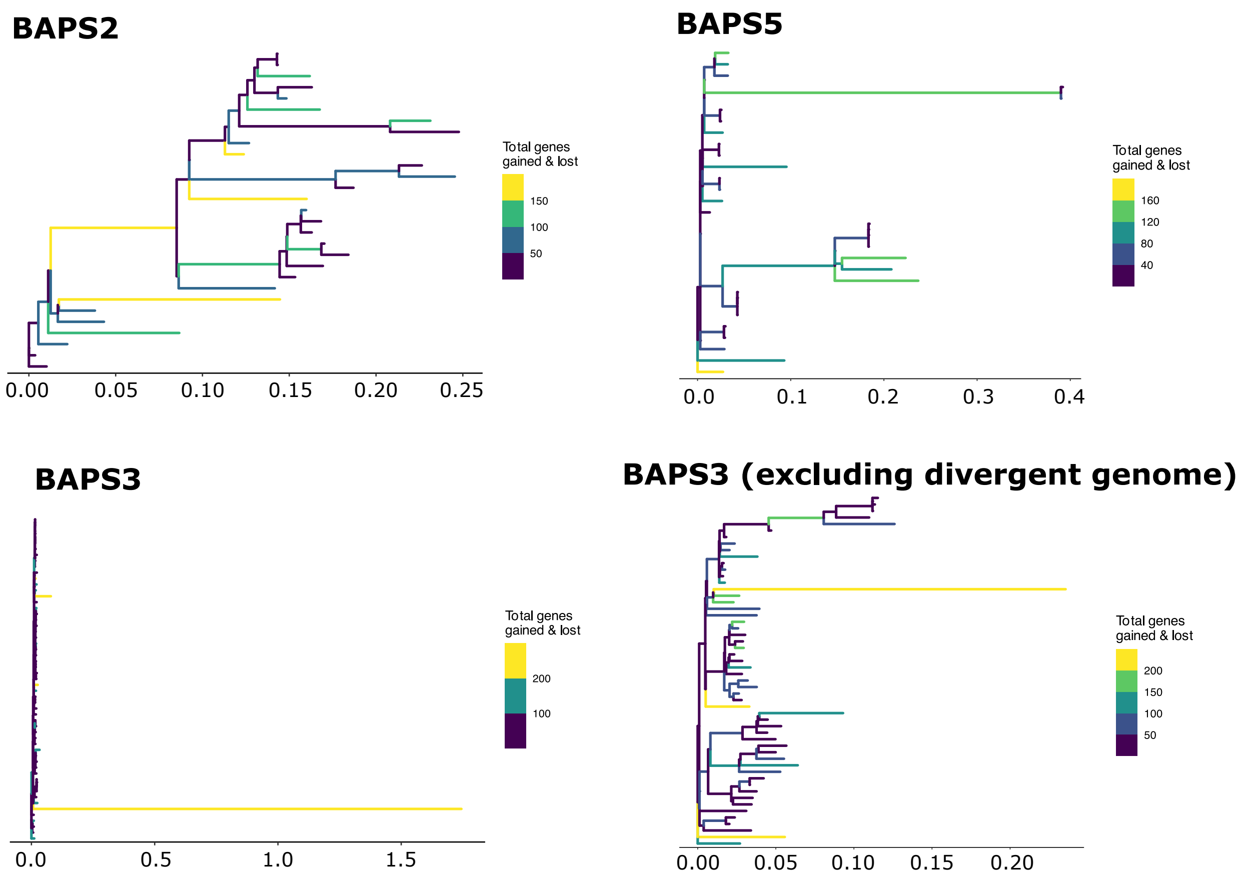

Supplementary Figure S2. Inferred ancestral states showing the gene gain and loss events on phylogeny of sequence clusters BAPS2, BAPS5, BAPS3, and BAPS3 excluding the divergent genome (Accession no. GCF_003609855.1). The branch colors show the total number of genes gained and lost along the internal and terminal branches of the trees.
